# Editorial: New insights into cancer immunology of refractory GU and liver tumors: therapy and mechanism

**DOI:** 10.3389/fimmu.2024.1420381

**Published:** 2024-05-01

**Authors:** Miao Liu

**Affiliations:** Department of Pathology, Brigham and Women’s Hospital, Harvard Medical School, Boston, MA, United States

**Keywords:** cancer, cancer therapy, cancer immunology, mechanism, PROTAC

Over the past few years, research in cancer immunology has made leaps and bounds, offering us novel approaches to treating various solid tumors—particularly those of the genitourinary (GU) system and the liver, which have been challenging to treat. Some immunotherapy treatments have achieved a degree of success in certain contexts and the advent of immune checkpoint inhibitors, such as PD-1/PD-L1 and CTLA-4 inhibitors, has brought new hope for the treatment of these intractable tumors (1). These therapies work by unblocking the immune suppression of anti-tumor T cells, leading to T cell multiplication and an enhanced anti-tumor response. Specifically, PD-1/PD-L1 inhibitors disrupt the interaction between PD-1 on T cells and PD-L1 on tumor cells, reactivating immune cells to attack the tumor. This mechanism has been explored in various studies, demonstrating the efficacy of peptide-based inhibitors and small molecules targeting PD-1/PD-L1 pathways. For instance, the development of peptide-based PD-1/PD-L1 inhibitors, like AUNP-12, has shown promising results in animal studies by effectively inhibiting tumor cell growth and metastasis with minimal adverse reactions (2). The clinical application of PD-1 inhibitors like Nivolumab and Pembrolizumab for treating hepatocellular carcinoma (HCC) has shown promise, as evidenced by various studies. One multicenter study demonstrated the clinical activity of Nivolumab in patients with advanced refractory biliary tract cancer, showcasing a disease control rate of 59%. This highlights the potential of immune checkpoint inhibitors (ICIs) in managing cancers that are traditionally hard to treat. Furthermore, a real-world comparison study of Nivolumab and Pembrolizumab in patients with advanced HCC suggested no significant differences in survival outcomes between the two, underlining their potential utility in HCC treatment (3). These findings reflect the evolving landscape of immunotherapy in treating liver cancers, especially HCC and cholangiocarcinoma (ICC), where ICIs play a central role. However, challenges such as drug resistance and low efficacy in some patients highlight the need for further research and development of more effective treatment strategies (4). Emerging technologies, such as protein degraders, may offer potential solutions to these problems. Existing Targeted protein degradation (TPD) technologies, such as PROTACs or molecular glues, are largely based on small molecules structures, making the selection of small molecule ligands for current immune checkpoint proteins challenging. Therefore, the emerging Peptide-based PROTAC technology, which can leverage protein-protein interactions to more easily select relevant ligands for degrading immune checkpoint molecules, holds promise. Structurally optimized peptide-based PROTACs achieving nanomolar DC50 values lay a solid foundation for their potential clinical translation in the future Shi et al.

A key challenge in the treatment of refractory cancer is the potential impact of inter-individual genetic differences on treatment outcomes. Recent studies have highlighted the critical role of genetic background in tumor genesis, progression, and response to specific treatments. This insight profoundly affects the personalization of cancer treatment, especially for tumors exhibiting significant variability in response to conventional therapies (5, 6). Patients from different ethnic backgrounds may exhibit varying levels of efficacy and tolerance to certain immune checkpoint inhibitors, in part due to genetic variations that affect immune regulatory pathways. Thus, understanding a patient’s genetic background becomes crucial when devising treatment plans. The wealth of existing databases containing patients’ gene expression profiles and clinical data forms a significant basis for more accurate treatments and early diagnosis. By analyzing gene expression profiles and clinical data from patients with clear cell renal cell carcinoma (ccRCC) in the Cancer Genome Atlas (TCGA), it is possible to identify lncRNA types associated with tumor progression. This enables the development of models for early diagnosis and prognostic prediction in ccRCC. Discovering significant correlations between ESTIMATE scores, tumor mutation burden, and tumor stemness indices with the disease risk score highlights differences in immune function and cell infiltration across various risk groups. Additionally, this provides potential guidance for personalized treatment strategies for ccRCC in the future Zhang et al.

In the field of cancer immunology research, identifying new targets and understanding how signaling pathways influence treatment outcomes have become crucial for achieving therapeutic breakthroughs. Besides the PD-1/PD-L1 pathway, kinase targets, Hepatocyte Growth Factor Receptor (VEGFR), YAP/TEAD, c-Myc, etc., all play key roles in the determination of cell fate, proliferation, and cancer development in HCC. Furthermore, targeted therapy against tumor-specific antigens (TSA) has been considered a crucial approach in cancer immunotherapy. GPC3 and SALL4, both oncofetal protein biomarkers, have been found to be aberrantly expressed in a variety of cancers, especially in the context of targeting hepatocellular carcinoma Liu et al. Their overexpression is closely associated with tumor aggressiveness, poor prognosis, and chemotherapy resistance, yet they are minimally expressed in normal cells, making them attractive therapeutic targets. Therapeutic strategies targeting these TSA targets like SALL4 can disrupt tumor cell proliferation and survival signals, thereby inhibiting tumor growth and spread (7). The combination of SALL4 with PD-1/PD-L1 inhibitors is anticipated to become a new avenue for enhancing the effectiveness of tumor immunotherapy.

The continuous advancements in cancer immunology have profoundly impacted the treatment of tumors. By delving deeper into the new biomarkers and signal pathways immune evasion, it is anticipated that more effective, targeted therapies with fewer side effects will be developed, offering more treatment options and hope to patients with refractory GU and liver tumors. Moreover, personalized treatment strategies based on the specific genetic background, tumor characteristics, and immune environment of the patient will contribute to achieving optimal treatment outcomes.

## Author contributions

ML: Conceptualization, Writing – original draft, Writing – review & editing.
